# Present State of the Art of Composite Fabric Forming: Geometrical and Mechanical Approaches

**DOI:** 10.3390/ma2041835

**Published:** 2009-11-17

**Authors:** Abel Cherouat, Houman Borouchaki

**Affiliations:** Charles Delaunay Institute-Project GAMMA3, University of Technology of Troyes, 12 rue Marie- Curie, BP 2060, 10010 Troyes, France; E-Mail: houman.borouchaki@utt.fr (H.B.)

**Keywords:** composite fabric, finite deformation, geometrical approach, FEM, draping and deep-drawing processes

## Abstract

Continuous fibre reinforced composites are now firmly established engineering materials for the manufacture of components in the automotive and aerospace industries. In this respect, composite fabrics provide flexibility in the design manufacture. The ability to define the ply shapes and material orientation has allowed engineers to optimize the composite properties of the parts. The formulation of new numerical models for the simulation of the composite forming processes must allow for reduction in the delay in manufacturing and an optimization of costs in an integrated design approach. We propose two approaches to simulate the deformation of woven fabrics: geometrical and mechanical approaches.

## 1. Introduction

Composite materials with glass, carbon or aramid fibres and polymeric matrices are known to have high specific stiffness and, in combination with automatic manufacturing processes, make it possible to fabricate complex composite structures with high levels of weight and cost efficiency. As known, the substitution of metal alloys by composite materials, in general, reduces structure mass by 20–30%. This increase is also due to the wide variety of semi-products (roving, fabrics, knitted fabrics, braids pre-impregnated or not) available permitting the elaboration of new structures. Fabrication processes, also, have undergone substantial evolution in recent years. Although the traditional lay-up process will remain the process of choice for some applications, new developments in resin transfer moulding, sheet moulding compound, low temperature curing prepregs and low pressure moulding compounds are reached new heights of sophistication, and are now being exploited in high technology areas such as the aerospace industry [1,2,3,4,5].

The simulation of the manufacturing of a textile reinforced composite part with a Liquid Composite Moulding-like process, which involves draping (or deep-drawing) and impregnation of the preform, includes several stages. First, a mould is designed with CAD software, and the CAD model is meshed. Then, a draping (or deep-drawing) simulation tool is used to compute the deformations of the textile layers inside the mould. As a result, for every element of the mesh, textile parameters like the shear angle and the thickness of the layer are available. With these parameters given, the local (meso-scale) properties for every element is determined (pre-processing), and given as input for the macroscopic structure after resin polymerization simulation. The result of the macro-simulation is then post-processed to optimize the mechanical properties of composite structure. The numerical simulation of composite forming is an efficient means of evaluating factors related to manufacturing processes and an efficient help to design pre-forming sequence for the manufacturing of fabric reinforced composites. It is thus possible to detect the main problems occurring during the shaping deformation and to obtain good quantitative information on the forming process [4,5,6,7,8,9,10].

**Figure 1:**
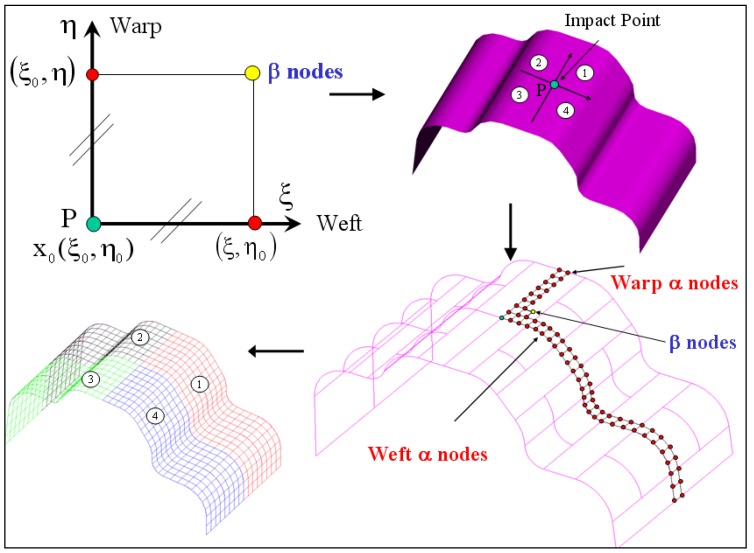
Geometrical draping of composite fabric.

The choice of manufacturing process depends on the type of non-polymerized matrix (or resin) and fibres, the temperature required to form the part and the cost effectiveness of the process. Often, the manufacturing process to be used is the initial consideration in the design of composite parts. Each manufacturing process imposes particular limitations on the structural design. It is necessary to develop specific algorithms taking into account not only the appropriated mechanics of the semi-products but also the associated process. Different levels of modelling intervene in the design process (see Figure 1): architecture design level, pre-dimensioning level, mechanical level by computational software and optimisation level [11,12,13,14,15,16,17,18,19].

Laying-up or draping is the most common method of producing thin composite parts [14,20,21,22]. The primary methods of automation in hand lay-up relate to computer software. Software is used to generate flat patterns from the layer surface and the ply boundary and to find the most efficient nest of cut plies to minimize the scrap. The formulation of the more efficient numerical models for the simulation of the forming composite processes must delays in manufacturing of complex parts and an optimisation of costs in an integrated design approach. The composite manufacturing process involves large displacements and rotations and large shears of weft and warp fibres, which can have a significant effect on the processing and structural properties of the finished product. The formulation of new and more efficient numerical models for the simulation of the shaping composite processes must allow for reduction in the delay in manufacturing of complex parts and an optimization of costs in an integrated design approach [3,23,24,25].

In this study, we propose two numerical approaches to simulate the forming of composite fabrics: geometrical and mechanical approaches. The geometrical approach is well adapted to pre-dimensioning level. It is based on geometrical aspects of the warping. Our method is based on a modified "MOSAIC" algorithm, which is suitable to generate a regular quad mesh representing the lay-up of the curved surfaces (giving the exact fibre orientations). The method is implemented in the GeomDrap software [26] which is now integrated in the ESI-Pam software. This software provides a fibre quality chart (showing the fibre distortions, the rate of falling and the rate of draped surface) to predict difficult impregnated regions. It can be used to optimise the draping process (with respect to the above quality measure) by improving the lay-up directions or the marker data location. The lay-up of complex curved surfaces can be made in a few seconds [12,16,27,28,29]

The mechanical approach is described on a meso-structural approach for finite deformations and geometrical non-linearity. The not polymerized resin has a viscous behavior and the reinforced fibers are treated as either unidirectional non linear elastic behavior. The bi-component finite elements for modeling composite fabric behavior are based on an association of 3D membrane finite elements representative of resin behavior and truss finite elements representative of warp and weft fibers behavior. The efficiency of the proposed model resides in the simplicity of its finite element discretization and the performance of its mechanical background [10,12,30,31,32,33,34,35,36,37,38].

However, due to the imposition of large displacements, the bi-component finite element mesh representing the workpiece undergoes severe shear of fibres hence, necessitates remeshing or the generation of a new mesh for the deformed/evolved geometric representation of the computational domain [39,40,41,42,43,44]. It is therefore necessary to update the mesh in such a way that it conforms to the new deformed geometry and becomes dense enough in the critical region while remaining reasonably coarse in the rest of the domain. In this paper we give the necessary steps to remeshing a mechanical composite structure subjected to large displacement. An important part is constituted by geometric and physical error estimates.

## 2. Results and Discussion

### 2.1. Geometrical approach

In this section, we present the mathematical formulation of the geometrical draping of composite fabric and then we propose a new algorithm scheme to solve the numerical draping problem. Let us denote by Σ the non developable tool surface of the part to drape and we assume that a geometrical mesh $T_{\Sigma }$ of this surface is known. Let Φ be the woven composite fabric modelled by two orthogonal and inextensible fibre families (warp and weft) described by the local coordinates x=(ξ,η). These families constitute regular quadrilateral fabric mesh called $T_{F}$ of the fabric Φ (Figure 1). The problem of geometrical draping of Φ onto the non developable surface ∑ consists of calculating displacement of each connecting point of warp and weft with a point of the tool surface mesh $T_{\Sigma }$ such that the lengths of the edge of the corresponding mesh $T_{F}^{\Sigma }$ on the surface are preserved (no extensible of fibre). This problem is nonlinear and presents infinity of solutions depending on two parameters:

- Starting point associated with a node of fabric $T_{F}^{\Sigma }$.
- Initial warp and weft orientation α.

To ensure a unique solution, we suppose that the starting points (corresponding to the point of impact of the machine to drape) $x_{0}^{\Sigma }$ on the tool surface as well as the fibre orientation α are given. The draping algorithm scheme is given by the following steps [27]:

- Associate the starting point on geometrical part mesh of the mould $x_{0}^{\Sigma }=\left(\xi_{0},\eta_{0}\right)$.
- Compute numerically step by step the warp nodes of the corresponding fabric mesh $T_{F}^{\Sigma }$, along the geodesic line defined by orientation α, classified as α-nodes, from the starting point, associated with nodes $\left(\xi ,\eta_{0}\right)$ of quadrilateral fabric mesh $T_{F}$.
- Compute numerically step by step the weft nodes of the corresponding fabric mesh $T_{F}^{\Sigma }$, along the geodesic line defined by orientation (α+90°), classified also as α-nodes, from the starting point, associated with nodes $\left(\xi_{0},\eta \right)$ of quadrilateral fabric mesh $T_{F}$.
- Compute numerically cell by cell all the other nodes of $T_{F}^{\Sigma }$, classified as β-nodes, from *x*_0_ and the nodes associated with nodes $\left(\xi ,\eta_{0}\right)$ and $\left(\xi_{0},\eta \right)$ of quadrilateral fabric mesh $T_{F}$.

The nodes of $T_{F}^{\Sigma }$ associated with nodes $\left(\xi ,\eta_{0}\right)$ and $\left(\xi_{0},\eta \right)$ of quadrilateral fabric mesh $T_{F}$ (the α-nodes) are put on the tool surface along the geodesic lines emanating from the starting point. Regarding the β-nodes, various algorithms are proposed [29,45,46,47,48]. Most of them use an analytical expression of the surface and formulate the draping problem in terms of non-linear partial differential equations. Other algorithms are also proposed to simplify these equations by using a discrete approximation of the surface by flat triangular face (*i.e.*, a mesh of the surface). Based on this latter approach, we propose a new algorithm in which the geodesic lines on the surface are approximated by the polylines plotted on the surface using linear orthogonal transformation in ℜ^3^ to setting flat the surface locally (these polylines become a straight line segment after these transformations). This allows us to determine the α-nodes. The β-nodes are computed by solving an optimization problem corresponding to determine a vertex of an equilateral quad plotted on the surface from the data of the three other vertices. This optimization problem formulates the direction of the geodesic lines emanating from the searched vertex [2,19,27,32,45,47].

### 2.2. Mechanical approach

**Figure 2:**
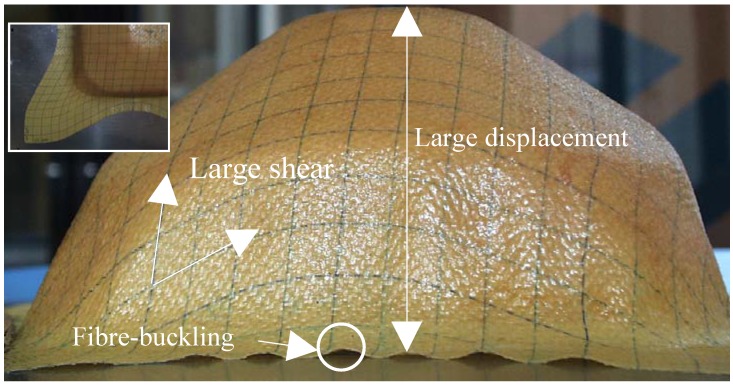
Finite deformations of composite fabric.

The deformation modes of composites fabrics during the forming process are different than those of sheet metal [10,17,49,50,51,52,53,54,59]. A number of deformation mechanisms are available, including shear deformation between warp and weft fibres, fibre straightening, relative fibres slip and yarn buckling. In the deep-drawing or the draping processes of composite fabrics, the evolution of a two straight line grille drawn alternatively on warp and weft fibre directions, shows that the lines become curved but remain continuous (see Figure 2). The absence of inter-yarn sliding (ensured by the weaving fabric, viscoelastic behavior of non-polymerized matrix, friction fiber/fiber and fiber/matrix) can be observed over the main areas of the fabric (*i.e.,* far enough from the free edges of the fabric). Also, for the composite fabrics based on high modulus, the compressive as well as bending stiffness are negligible compared to the in-plane membrane stiffness. The assumption is that each cross connexion of straight warp and weft fibre before deformation remains cross connected during the transformation. The basic assumptions for the mechanical forming are that the woven fabric is considered as a continuous 3D surface. The warp and weft fibres are assimilated as a truss which connecting points are hinged and the membrane resin is coupled kinematically to the fabric at these connecting points.

**Figure 3:**
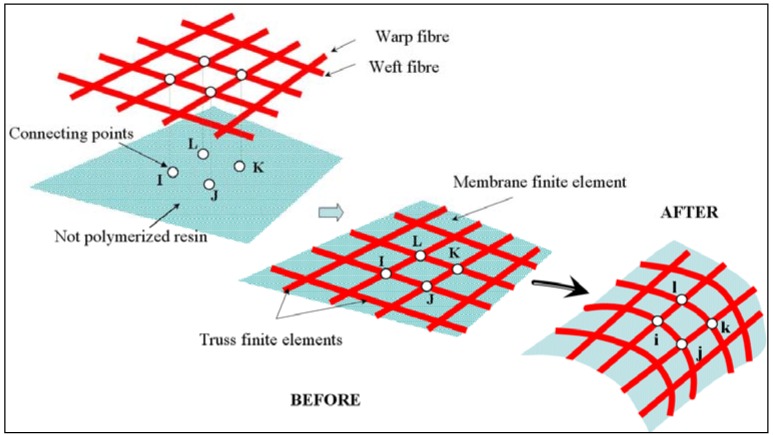
Mechanical deformation of composite fabric.

Each fibre (warp and weft) point ${\vec{X}}^{f}$ in the reference configuration coincides to resin point ${\vec{X}}^{m}$ (see Figure 3). At the connecting points we have ${\vec{X}}^{f}={\vec{X}}^{m}=\vec{X}$ before deformation. The current position of these points is obtained by:

(1) $$\{\begin{array}{l} \begin{array}{cc} d{\vec{x}}^{f}=F^{f}\left(\vec{X},t\right)d{\vec{X}}^{f} & \mathrm{fibres} \end{array}\\ \begin{array}{cc} d{\vec{x}}^{m}=F^{m}\left(\vec{X},t\right)d{\vec{X}}^{m} & \mathrm{re}\sin \end{array} \end{array}$$

where $F^{f}$ and $F^{m}$ are the deformation gradient tensor of fibre and resin respectively. The relationship of the no sliding inter-fibre can write at each connecting point as $\vec{x}=\vec{x}(\vec{X},t)$ with ${\vec{x}}^{f}={\vec{x}}^{m}=\vec{x}$. For these points we have $F^{f}=F^{m}$ but for all other point $F^{f}\neq F^{m}$. It is possible to decompose the deformation gradient tensor in terms of the rigid rotation tensor followed by a stretch. By the spectral theorem, the stretch component (longitudinal elongation of fiber $\lambda_{L}^{f}$ and tensor strain of resin $U^{m}$) is defined in the reference configuration C_0_ as :

(2) $$\{\begin{array}{ll} \lambda_{L}^{f}=\sqrt{N_{L}^{f}F^{f^{T}}F^{f}N_{L}^{f}} & \mathrm{fibres}\\ U^{m}=\sqrt{F^{m^{T}}F^{m}} & \mathrm{resin} \end{array}$$

Using the above assumptions, the mechanical behaviour of composite fabric depends on the relative movement of fibres and the deformation of resin. The problem of the integration of strain rate tensors is a central one in large deformations. The rate equations for finite strains use objective derivatives [55]. The frame associated with Green-Naghdi’s derivative is defined, at the material point considered, by the rigid body rotation of the triplet orthogonal material directions ($\left(N_{i}^{f}⊗N_{i}^{f}\right)$ for the fibre and $\left(e_{0i}⊗e_{0i}\right)$ for the not polymerized resin). The stretching tensors are written in the material frames:

(3) $$\{\begin{array}{l} \begin{array}{cc} {\bar{\lambda }}_{L}^{f}=\left(\frac{{\dot{\lambda }}_{L}^{f}}{\lambda_{L}^{f}}\right)\begin{array}{cc} \left(N_{L}^{f}⊗N_{L}^{f}\right) & \end{array} & \mathrm{fibres} \end{array}\\ \begin{array}{cc} {\bar{D}}^{m}={\dot{U}}^{m}U^{m^{-1}}\left(e_{0i}⊗e_{0i}\right) & \mathrm{resin} \end{array} \end{array}$$

The longitudinal fibres stress ${\bar{\sigma }}_{L}^{f}$, depending on the fibre stretching elongation ${\bar{\text{D}}}_{L}^{f}$, and the not polymerized resin stress tensor ${\bar{\sigma }}^{m}$ can be written at each time as:

(4) $$\{\begin{array}{ll} {\dot{\bar{\sigma }}}_{L}^{f}=E_{L}^{f}\left(\lambda_{L}^{f}\right){\bar{D}}_{L}^{f} & \mathrm{fibres}\\ {\dot{\bar{\sigma }}}^{m}=C^{m}\left(\tau \right):{\bar{U}}^{m} & \mathrm{resin} \end{array}$$

The behaviour in tension of the fibres is supposed elastic non-linear (due to the fabric weaving) and is related to the elastic modulus ${\bar{E}}_{f}$ and fibres undulation factor $\varepsilon_{\mathrm{sh}}$. The isotropic viscoelastic behaviour of resin is formulates in the time domain by the hereditary integral and using the relaxation time $\tau_{k}$ and the fourth order relaxation tensor, which are material parameters $C_{ij}^{m^{k}}$:

(5) {ELf(λLf)=E¯f(1+exp(−λ˙LfλLfεsh))fibresCijm(t)=Cijm∞+∑1kCijmke−tτkresin

Each material point is moving as in a continuum, ensured by the non-sliding of fibers due to fabric weaving and resin behavior (see Figure 2). Therefore, a nodal approximation for the displacement can be used. The deformation of composite fabric is described within the frame of membrane assumptions. The energy of deformation is obtained by a summation of membrane strain energy of not polymerized resin and elastic tensile strain energy of fibers as:

(6) $$\delta \Pi \left(\dot{u}\right)=h_{0}\int_{\Gamma^{m}}{\bar{\sigma }}^{m}:\delta {\bar{D}}^{mR}d\Gamma +S_{0}^{f}\sum_{\mathrm{fibres}}\int_{L^{f}}{\bar{\sigma }}_{L}^{f}:\delta {\bar{D}}_{L}^{f}ds-\int_{\Gamma_{\sigma }}\bar{t}.\delta \dot{u}d\Gamma -\int_{\Gamma_{c}}{\bar{t}}_{c}.\delta \dot{u}d\Gamma$$

where $S_{0}^{f}$ and $h_{0}$ denote respectively, the initial cross section of the fibre and the thickness of the membrane resin, $\bar{t}$ is the external load applied on the composite fabric surface $\Gamma_{\sigma }$ and ${\bar{t}}_{c}$ is the contact force between the mould tool and the fabric on $\Gamma_{c}$.

The effect of spatial equilibrium of composite material on the actual configuration is established in terms of nonlinear equations: kinematic non-linearity, material non-linearity and contact with friction non-linearity. It is linearized for each load increment by an iterative Newton-Raphson method. According to the different modes of deformation occurring in the composite fabric during the shaping process, bi-component finite elements are used to characterize the mechanical behavior of thin composite fabric before non-polymerized matrix. The bi-component element is based on an association of linear membrane finite element (Triangular and Quadrilateral) combined with a complementary truss linear finite element. The global stiffness of composite fabric is obtained by discreet summation of fibres (warp and weft) and not polymerized matrix stiffness. The governing equilibrium nonlinear equations (6) are solved using dynamic explicit integration. This approach has proven to be, in particular, suitable to highly non-linear geometric and material problems and where a large amount of contact between different structural parts occurs [12,19,56]. The Dynamic Explicit DE algorithm available in Abaqus/Explicit for solving the algebraic system works by using the lumped form of the mass matrix [57].

### 2.3. Remeshing scheme for composite forming

Let us recall that the adaptive remeshing of fabrics during draping of deep-drawing is never approached in the literature. In this work, we propose specific algorithm based on an iterative step to simulate numerically the forming process of composite fabric using commercial software ABAQUS. At first, a coarse initial mesh of the fabric is generated with bi-component finite elements (linear quadrilateral element representing the non-polymerized resin and linear truss elements representing the warps and the weft fibers). Then, remeshing procedure is applied after each displacement according to the following scheme:

- Definition of a physical size map based on the adaptation of the mesh element size with respect to one of the mechanical fields.
- Definition of a geometrical size map based on the geometric curvature of the boundary.
- Threshold refinement: numerical values of the estimated local error are used both to select the elements to be refined and to decide into how each new elements may be subdivided.
- Refinement with solution estimation: a solution approximation and its local error on a mesh are estimated from a previous approximation solution.

#### 2.3.1. Definition of a physical size map

A physical size map is defined by calculating a physical size for each element of the part. This physical size is defined with respect of one of the mechanical field. In this paper, the shear stress of the not polymerized resin has been chosen. Minimal element size is associated to maximal shear stress (given by the user from experimental tests) and a maximal element size for minimal shear stress and for the other elements, a linear size variation can be used. For a given element, if the ratio between the average size of its edges and its physical size is greater than a given threshold, the element must be refined.

#### 2.3.2. Definition of a geometrical size map

The geometrical size map indicates if a boundary element must be refined or not. The geometric curvature is estimated at each boundary vertex of the domain. If this curvature has been modified during the deformation of the computational domain and exceed a value limits given by the user (two degrees in our case), all elements sharing this boundary vertex must be refined.

The adaptive remeshing technique consists in improving the mesh by coarsening and refinement methods in order to conform to the geometry of membrane and truss elements during deformation. Four consecutive steps are executed:

- Coarsening step during which the mesh is coarsened with respect to the physical size map,
- Refinement step during which the mesh is refined according to the geometrical size map and then to the physical size map
- Define the deformed truss finite elements representing the fixed warp and weft fibres discretization.
- Transfer the mechanical field of non-polymerized resin (stress) and fibres (tensile force) from the old mesh to the new mesh.

**Figure 4:**
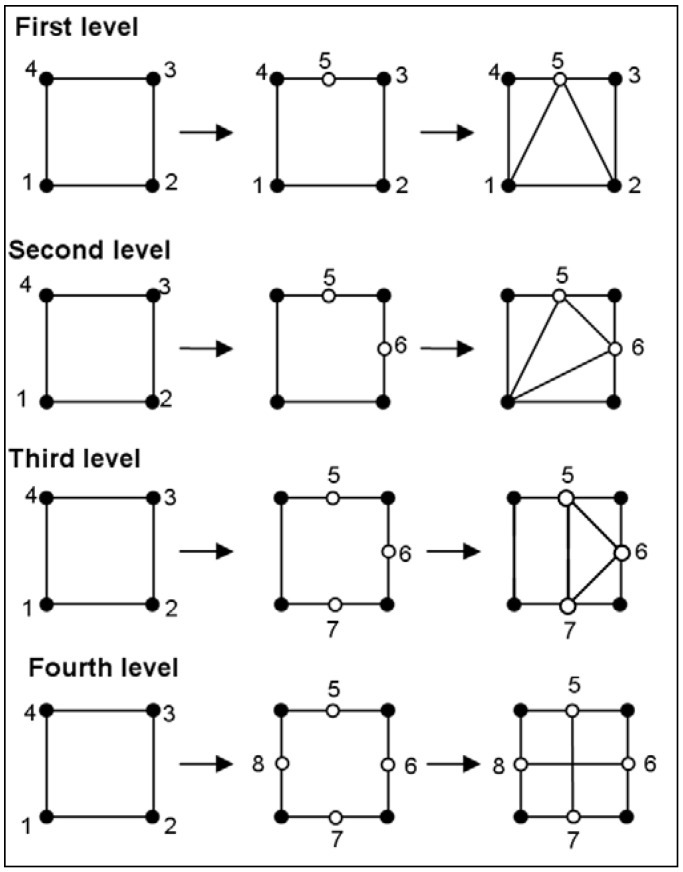
Different levels of fabric remeshing procedure.

There is only one element subdivision which allows preserving the element shape quality: the uniform subdivision into four new elements. For this subdivision, a node is added is the middle of each edge of the element. Boundary elements which belong to the geometrical size map are first refined. The refinement is then applied according to the physical size map. In this case, the refinement procedure is repeated as long as the physical size map is not reached. After each refinement procedure (geometrical criterion or physical criterion), an iterative refinement to restore mesh conformity is necessary. Indeed, after applying the subdivision according to the geometrical or physical criteria, adjacent elements to subdivided elements must be modified (see Figure 4). A procedure of subdivision has been proposed for the adjacent elements in order to stop the propagation of the homothetic subdivision (see [43,44] for more information). Specific procedure is developed in order to conserve the initial fibre density during the coarsening and refinement steps.

## 3. Experimental

### 3.1. Uniaxial test

Due to the importance of the composite fabric behaviour on material formability, tensile test of pre-impregnated fabric is proposed in order to study the influence of fibre orientation, fibre undulations due to fabric weaving and resin behaviour. At processing, the impregnated fabric is idealised as a viscous material subject to the kinematic constraints of incompressibility and inextensibility in the fibre direction. The pre-impregnated fabric tested in this study was a satin 5 with aramid woven fabric. The fabric was impregnated with epoxy resin using a hot-melt pre-pregging process. A lower loading velocity will generate lower viscous forces at the intra-ply shearing of fabric. In this experiment, a displacement in the vertical direction is imposed at the moving extremity of the rectangular composite specimen with three layers of fibres (length = 150 mm, width = 30 mm and thickness = 2 mm). The mechanical properties of the pre-impregnated fabric have been experimentally determined by [62]. The mechanical properties of the not polymerized resin are: elastic modulus = 45 MPa, Poisson’s coefficient = 0.45 and the time relaxation is $\tau_{k}=15.50s$ (see Table 1).

In the numerical modelling, the behaviour of the resin is assumed to be isotropic viscoelastic and that of the fibre, be elastic linear or elastic non-linear. Exponential law is used to describe the geometrical undulation of fibres and Dirichlet Prony series is used to approximate the creep functions relaxation of the not polymerized resin (Equation 5) [57].

**Table 1:** Mechanical properties of the pre-impregnated composite fabric [62].

${\bar{E}}_{f}$ (MPa)	ε_sh_	ρ (g/cm^3^)
130,000	0.005	1,45
**time (s)**	0.01	0.1	1	10	100	1000
**Shear C_12_^mk^**	0.02332	0.023332	0.083509	0.11723	0.14423	0.178

**Figure 5:**
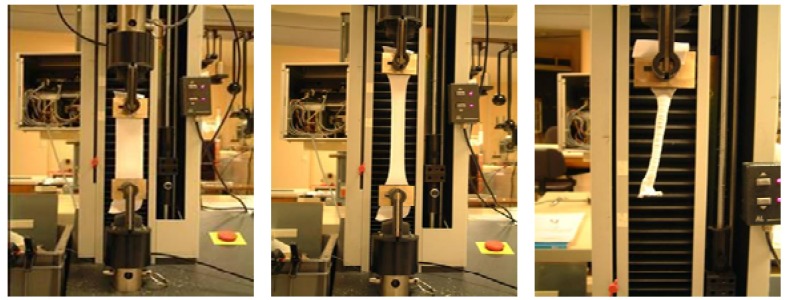
Tensile apparatus of pre-impregnated composite fabric specimen.

**Figure 6:**
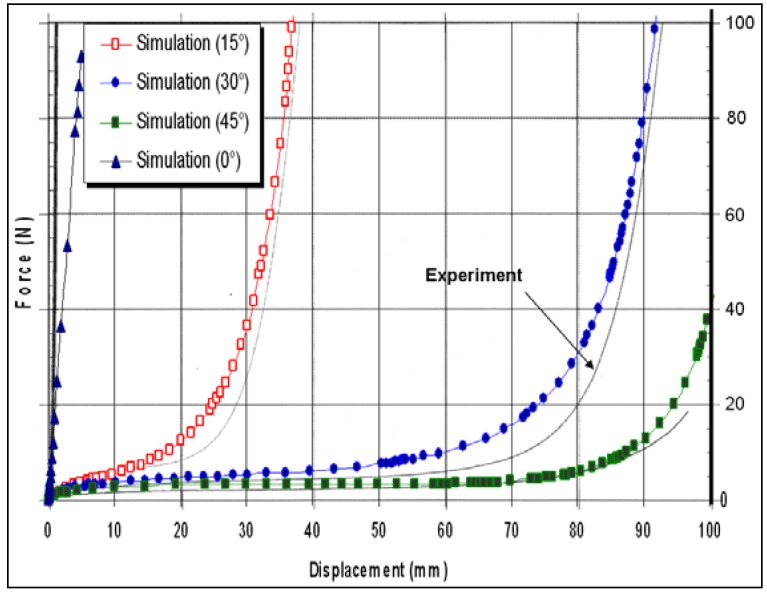
Load force- displacement of the fabric specimen for different fibre orientations.

The uniaxial tensile tests (Figure 5) are carried out for different orientations of fibres with the loading direction (0°, 15°, 30° and 45°). Four transformation phases are identified in the global response of the pre-impregnated woven fabric: viscoelastic phase resulting from not polymerized resin shearing behaviour (relaxation modulus), a pseudo-kinematic phase resulting from geometrical rotation of fibre and geometrical undulation of yarns, a hardening phase resulting from locking fibre, frictional resistance and from increasing density of fibres and linear phase resulting from fibre tensile behaviour. The experimental effort imposed by the tensile machine is compared to the numerical values for different fibre orientations in Figure 6.

**Figure 7:**
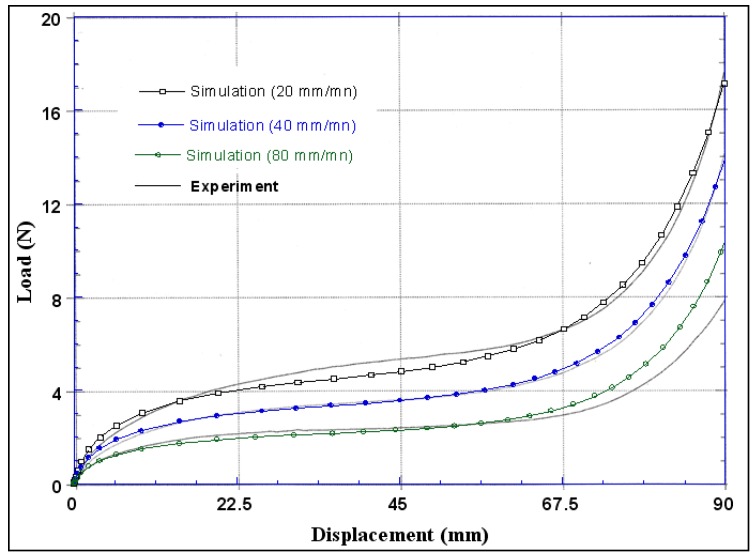
Effect of the load velocity of the global response for 45° of fibre orientations.

The effect of the imposed load velocities (20, 40 and 80 mm/mn) on the composite fabric for 45° of fibre direction is given in Figure 7. It should be noted that the load velocity influences considerably the first phase of the global response of pre-impregnated woven fabric (shearing behavior of the not polymerized resin). The behaviour of the not polymerized resin and the initial fibre orientation influenced largely the global response of the fabric during tensile loading. In this figure we can show the good correlation between the model and the experimental results [12]. Notice that the woven fabric material is highly anisotropic and the shrinkage factor of the woven fabric influences the total force and approaches better the experimental results. The numerical simulation agrees with the experimental results and proves the validity of the proposed model.

**Figure 8:**
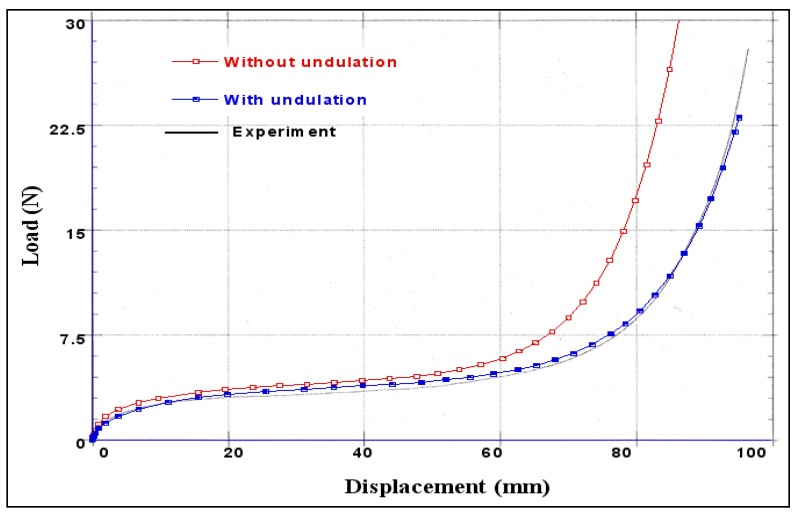
Effect of the fibre undulation of the force versus displacement response.

Figure 8 reports the experimental effort imposed by the tensile machine with respect to 45° fibre orientations and undulation variations ε_sh_ = 0 (the undulation effect is negligible) and ε_sh_ = 0.005. This figure describes, at the beginning of the curves, the viscoelastic phase followed by a kinematic stage which increases with the angle of loading. The end part of these curves represents the hardening phase with a very high stiffness due to fibre elongation. Notice that the woven fabric material is highly anisotropic and the initial directions of the fibre influence the final results.

The agreement between predicted and experimental values is good and proves the validity of the proposed model of pre-impregnated woven fabric behaviour. The numerical model described above clearly shows the strong non linearity of this behaviour law. It takes into account the mechanical characteristics of a viscoelastic resin, the anisotropic behaviour of fabric and the geometrical non linearities due to the high deformability of fibres (straightening and relative rotation).

### 3.2. Biaxial test

**Figure 9:**
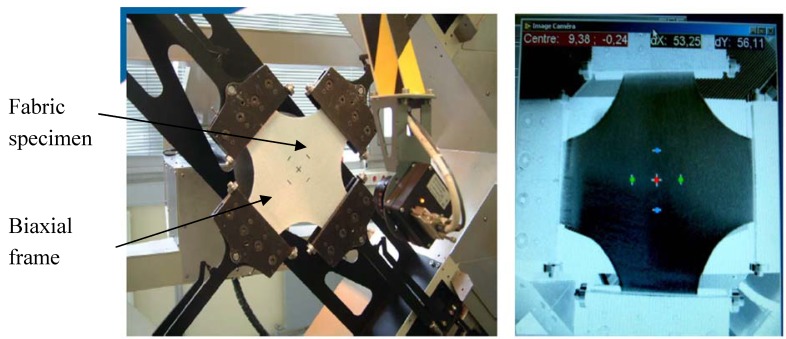
Device for a biaxial tensile test on cross fabric specimen.

In order to determine and analyse the undulation and interaction effects, a biaxial tensile apparatus (Figure 9) has been built in order to test woven materials in both the warp and weft directions [33,60]. For the realization of the biaxial tensile tests, we have used a triaxial dynamometer with five axis sensors of 20 kN capacities with a high frequency of recording (two along the X axis, two along the Y axis and one along the axis Z). Each half shaft can move in a range from 0 to 350 mm with a speed of up to 40 mm/s with the exception of half the Z axis is limited to a displacement of 200 mm. The loads are measured using load cells located very close to the specimen in order to avoid the influence of friction within the device. The shape of the specimen is square of dimension 250 × 250mm. To avoid stress concentration, rounding the specimen at the four corners with a radius of 50 mm is used. The effective length subjected to tension after the location of the specimen on the clamps is 200 mm in both directions X and Y. To determine the deformation in the central region of the specimen, it is a mark of nine points to identify the deformable zone. The camera can track the deformation of the specimen and the acquisition system can record the values of displacements in two directions in real-time [64]. The used balanced textile fabric (jersey) consisted of 77% polyamide fibres and 26% membrane elastane. The biaxial test is performed with a loading rate equal to 240mm/mn. To characterize the behavior of the used material from the overall response, we apply the inverse approach coupled with the optimization algorithm of Levenberg-Marquardt. The mechanical parameters of the polyamide fibres and the elastane membrane are given in [63].

**Figure 10:**
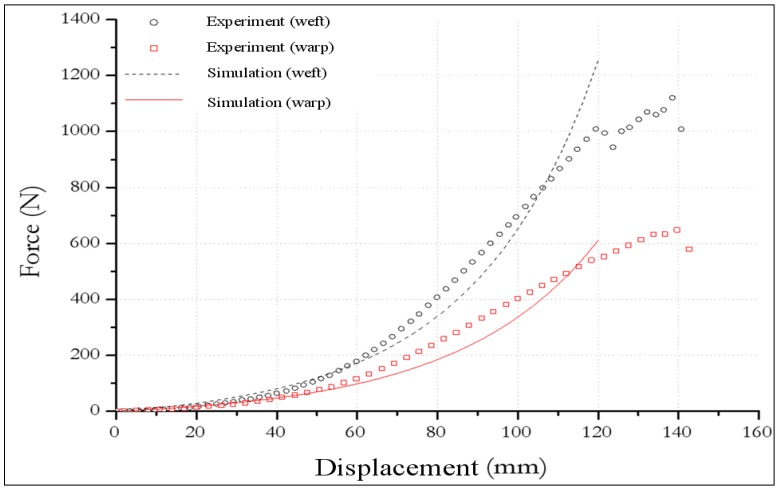
Tensile force versus displacement obtained from bias-extension tests.

**Figure 11:**
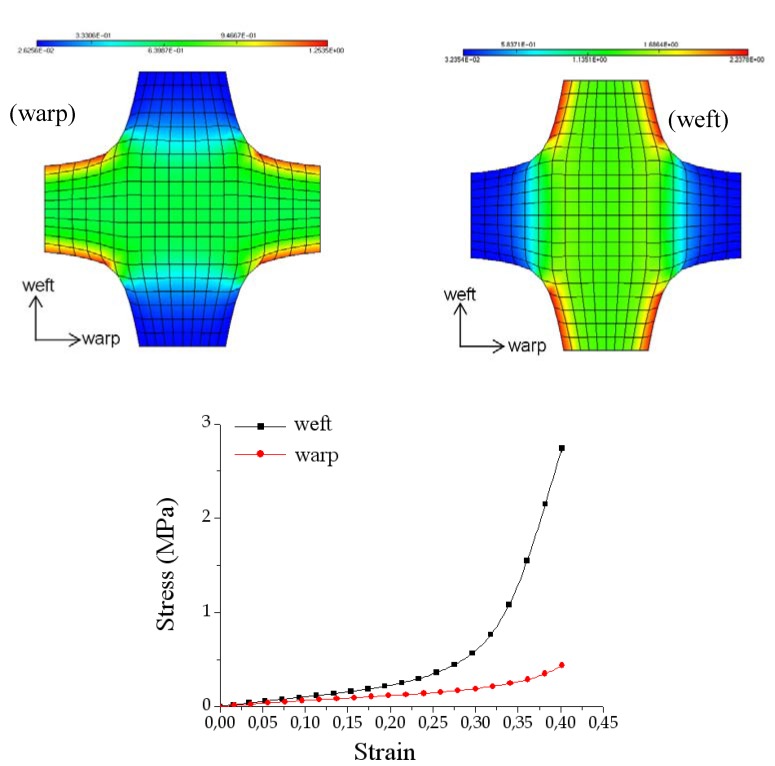
Contour of tensile force (a) warp direction and (b) weft direction.

For a given fabric, the response is strongly influenced by the strain ratio in warp and weft directions due to large fibre undulations. Especially, the non-linearity in warp and weft direction is increasing. The tendency of the fibre to straighten is more impeded when the strain in the perpendicular direction is large (see Table 2). The biaxial specimen, as shown in Figure 9, was modelled in 2D with 400 four nodes membrane elements (representative of elastic behaviour of elastane) and 1600 two nodes truss elements (representative of elastic non linear behaviour of polyamide fibres) of ABAQUS element library. The load was applied at the same time on each strip. The overall agreement of the proposed non-linear numerical model is very good in comparison with the experimental results (see Figure 10). The contour of the predicted stress in warp and weft fibre directions is illustrated in Figure 11. The maximum value of the stress in weft fibres is twice as large as in the warp direction.

**Table 2:** Mechanical parameters of the textile fabric.

	Warp direction	Weft direction
**Mass/mm^2^ (g)**	230	230
**Thickness (mm)**	0.744	0.744
**Number of fibre/cm**	64	64
**Section (cm^2^)**	0.16	0.16
**Maxi displacement (mm)**	140	140
**Maxi force (N)**	620	1180
**Maxi stress (MPa)**	0.45	2.65
**Maxi strain**	0.40	0.40

### 3.3. Deep drawing of woven composite reinforcements with conical tool

**Figure 12:**
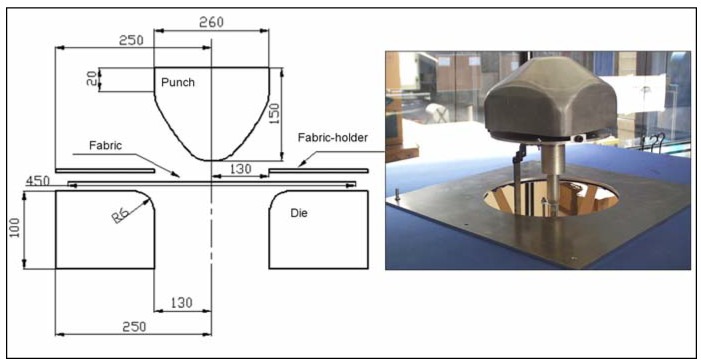
Geometry of deep-drawing tools.

**Figure 13:**
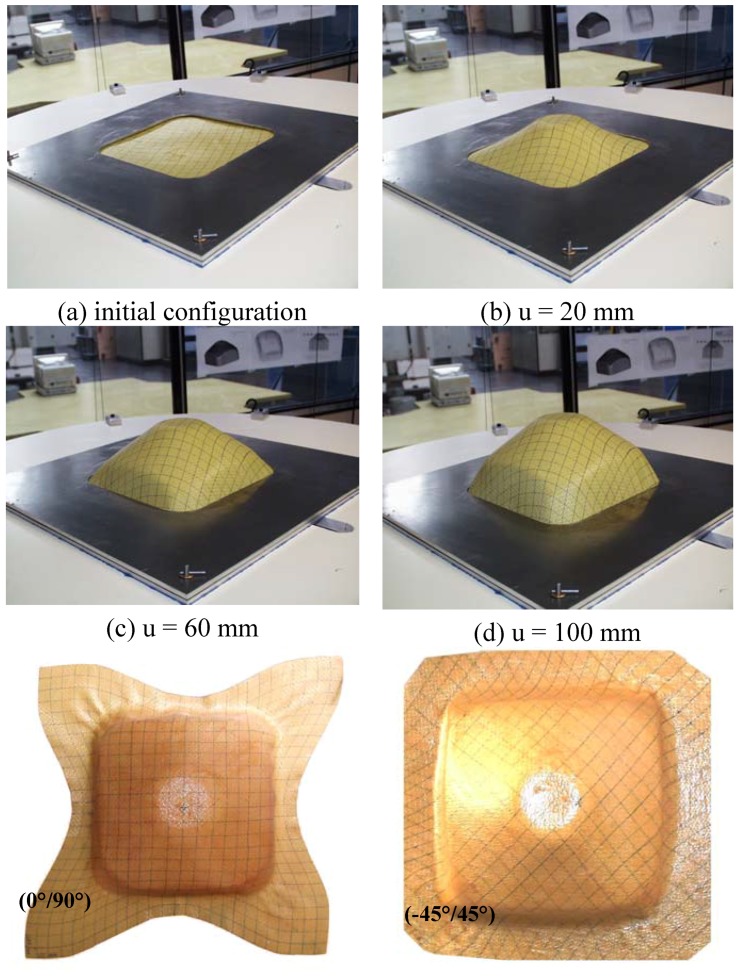
Experimental results (a) (0°/90°) and (b) (-45°/45°).

**Figure 14:**
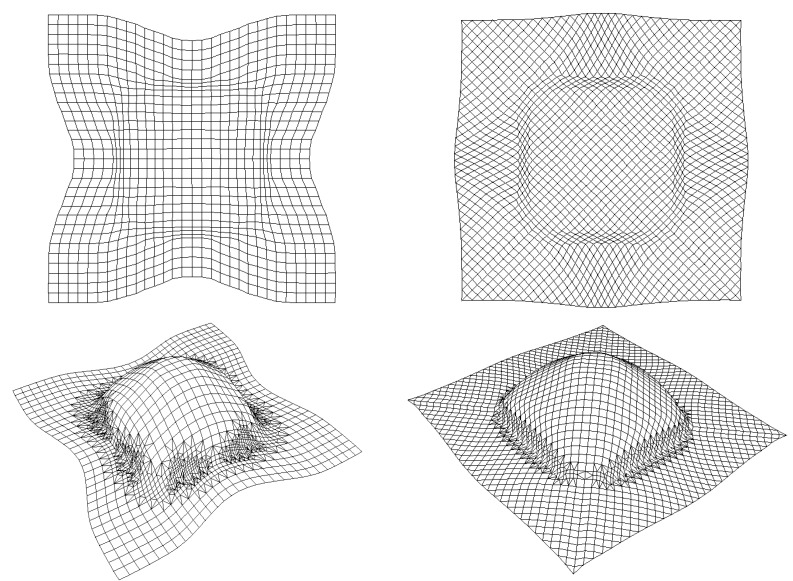
Numerical results with and without remeshing procedure.

The numerical analysis of composite fabric deformation by deep-drawing process is performed using the ABAQUS/EXPLICIT FEM-package [19,58]. The non-polymerized matrix is modelled by 1,600 linear membrane elements (M3D3) and the warp and weft fibres are modelled by 3,200 linear truss elements (T3D2). The mould tool is modelled by 1,600 linear rigid element (R3D3 or R3D4). The behaviour of the resin is assumed to be isotropic viscoelastic and the behaviour of the fibre is supposed as elastic (see Table 1). The example concerns the 3D deep-drawing of aramid pre-impregnated fabric with conical tools (see Figure 12). Figure 13 reports the experimental obtained shapes with respect to 0/90° and ±45° fibre orientation for different punch displacements (initial, u = 20, 60 and 100 mm). Experimental shapes and the corresponding final predicted shapes with and without remeshing procedure are shown in Figure 13 and Figure 14, respectively. The contour of the final shape after deformation is in good agreement with the experimental drawing. We can see that the initial thin fabric is computed using an initial coarse mesh, the mesh is again refined uniformly and the adaptive mesh refinement procedure is activated where elements are created automatically in regions of large curvature to even more accurately represent the complex material flow (large stretching) around the die radius.

**Figure 15:**
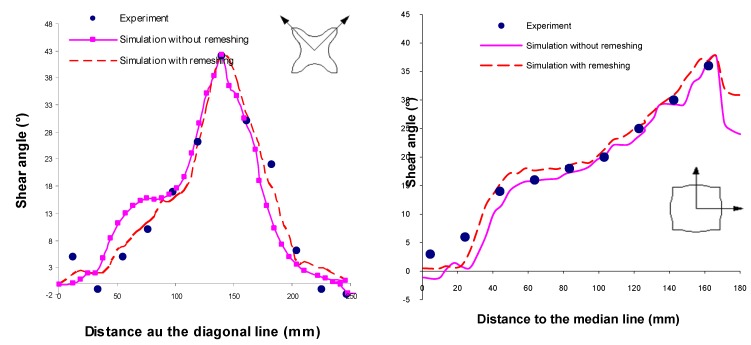
Shear variations along fabric lines with and without remeshing.

**Figure 16:**
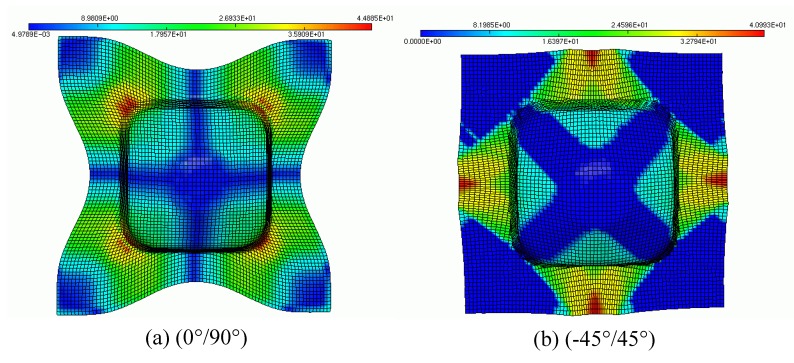
Iso-values of shear between warp and weft fibres.

The evolution of the predicted shear angle variations with and without remeshing procedure is compared in Figure 15 with the experimental values. We notice that these shear angle values are very large >38° along the median line for ±45° fabric and along the diagonal line for 0°/90° fabric. But along the median lines of 0°/90° and along the diagonal lines of ±45° the angular distorsions are very small <6°. Noting that, the remeshing procedure improves the predicted results in comparison with the experimental values. This result is confirmed by the iso-values result of shear angles between the warp and the weft fibres shown in Figure 16. On these figures, we notice the influence of the viscous effects of the resin behaviour on the final relative rotation of the warp and the weft fibre. A lower loading velocity will generate lower viscous forces at the intra-ply shearing. This will minimise the difficulty for a ply to conform to a given shape and should lead to less wrinkling.

### 3.4. Geometrical draping of complex CAD surface

**Figure 17:**
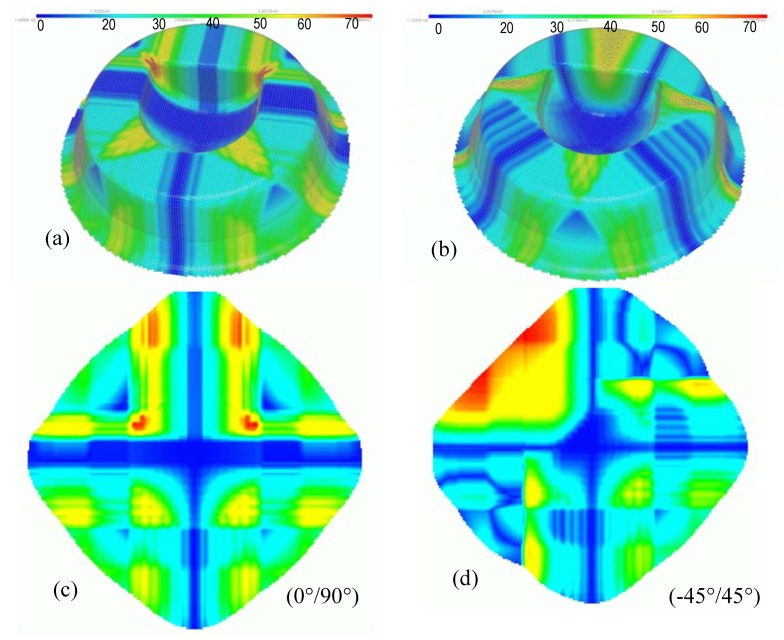
Draping of the CAD surface and corresponding 2D flat pattern.

We consider the geometrical draping of the surface corresponding to a complex CAD. The centroid of this piece is chosen as the starting point for which two different fibre orientations: (0°/90°) and (-45°/45°) are specified. Figure 17a and b show the draping as well as the fibre distortion of this surface. The corresponding 2D flat pattern (Figure 17c and d) associated with the fibre distortion map allows us to predict the location where the fabric must be eventually darted. One can notice that, in the considered cases, the surface of the piece is draped globally. However, in the second case, a smaller area of the flat fabric is used. This result shows the importance of the fabric orientation in the draping process.

### 3.5. Geometrical draping of hood car part

**Figure 18:**
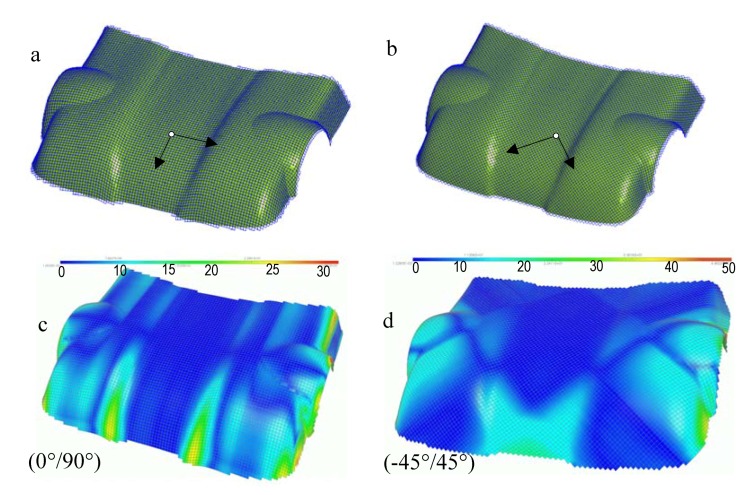
Geometrical draping of a car hood part and iso-values of shear angles.

The second example concerns the geometrical draping of a car hood. The centroid of the part is chosen as the starting point which the (0°/90°) and (-45°/45°) fibre orientations are specified. Figure 18a and b show the resulting 3D draping for the two orientations. We can note that all part surface is completely draped. Figure 18c and d present shaded contours interpolated from the map of the fiber distorsions of (0°/90°) and (–45°/45°) fiber orientations. The fiber distorsions for both (0°/90°) and (-45°/45°) draping are very small but the maximum shear angle localization are different (maximum shear angle is about 30° for (0°/90°) and 50° for (-45°/45°)).

### 3.6. 3D draping of textile fabric

**Figure 19:**
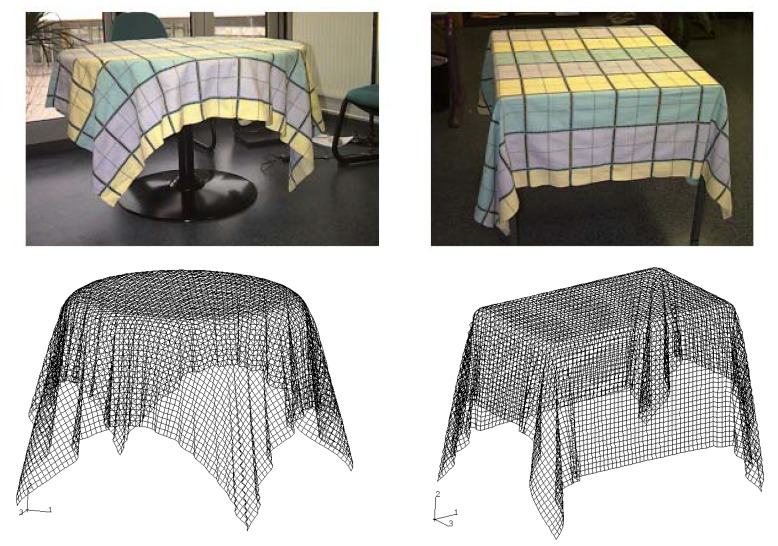
Experimental and numerical laying-up results of 3D textile fabric.

In this example, the draping of an initially plane square textile fabric (500 × 500 mm) over a circular or square table is considered. Figure 19 shows the three-dimensional experimental drape shapes of the fabric and the predicted shape using or no the remeshing method. The proposed approach gives a satisfactory shape after draping that involves large warp weft angles variations and wrinkles. It can be seen that these wrinkling are well described by the 3D membrane model. If some bending stiffness would be added, the wrinkling would be globally similar but they would be less numerous and larger as the bending stiffness increases (see [61] for more information). The draping is due to the weak shear stiffness and possible large warp weft angles variations. This again demonstrates the good drapability of the composite fabric studied here. Although qualitative comparison of experimental results, the present results give realistic shapes. The present method thus has potential for use in the development of powerful clothing CAD systems [19].

## 4. Conclusions

An efficient numerical approach (geometrical and mechanical with remeshing procedure) has been presented to simulate accurately the draping or deep-drawing of composite fabric. This numerical methodology is shown to be very helpful for composite forming process. An adaptive remeshing technique for composite fabric forming process with refinement and coarsening procedures has been proposed. The implementation with continuum triangular and quadrilateral elements and truss element in the ABAQUS code allows us to validate the proposed approach for several types of problems.
